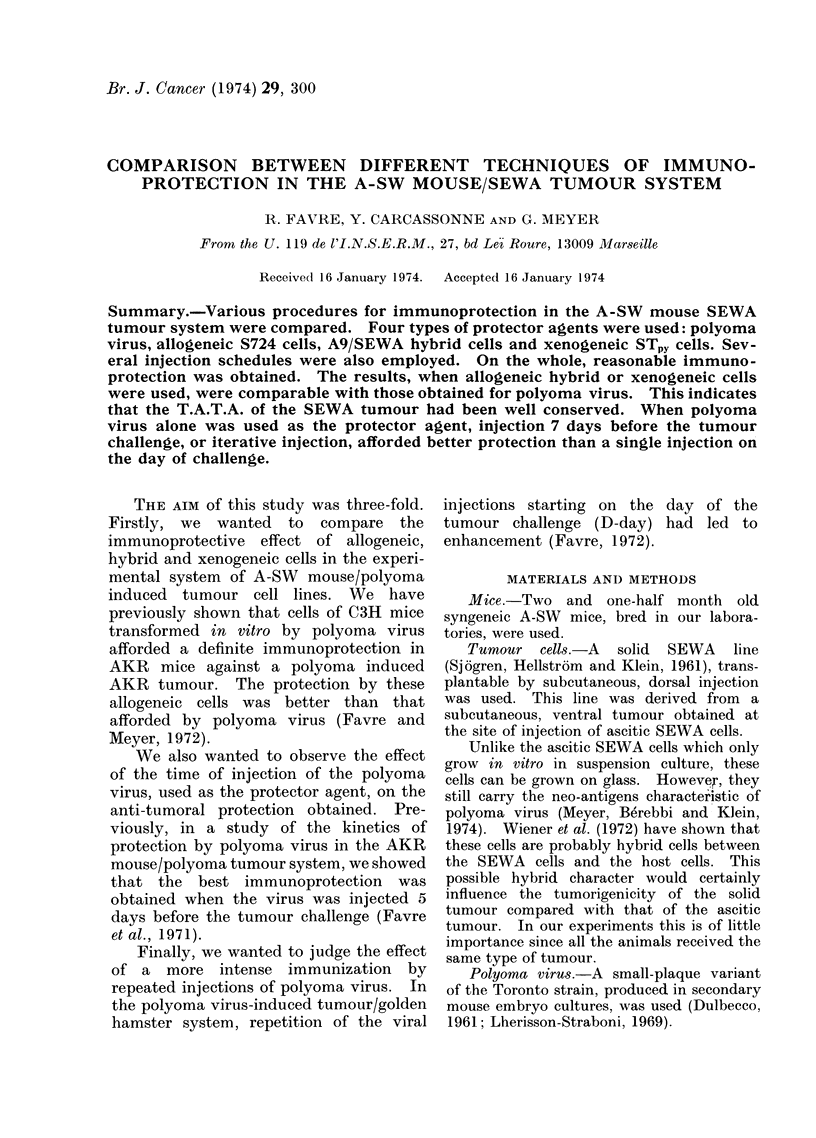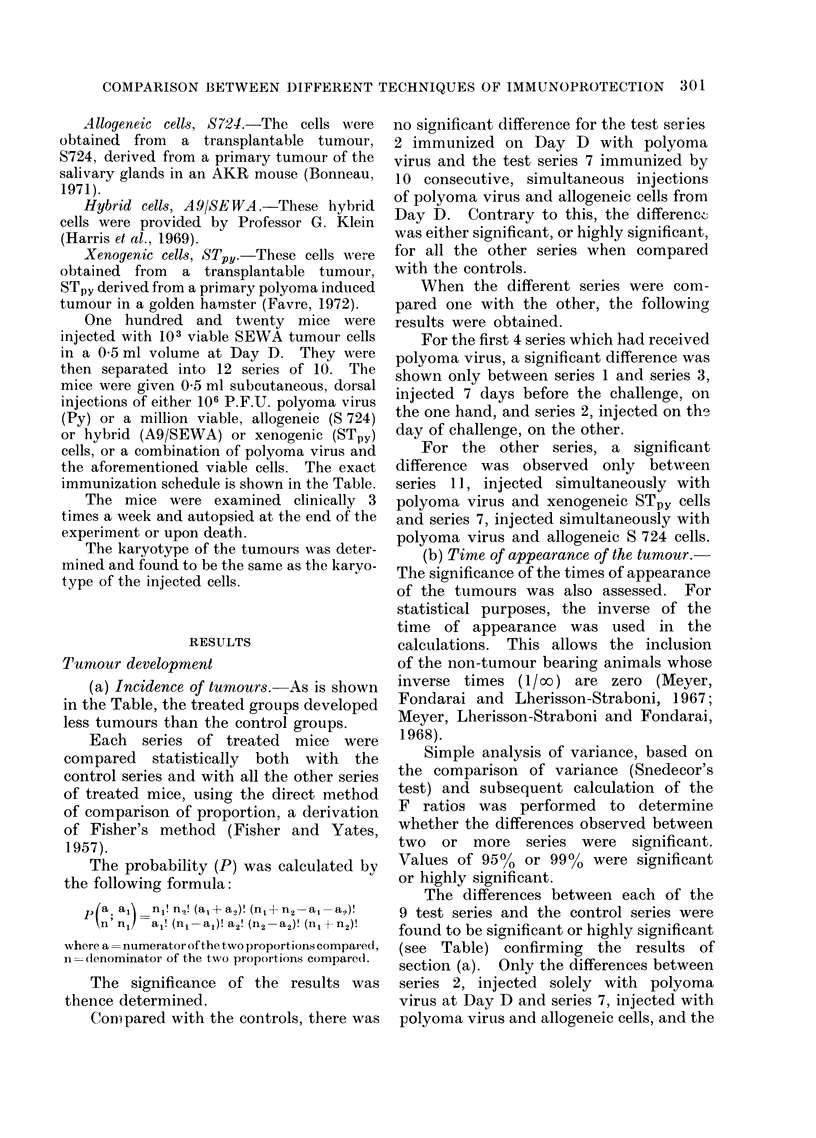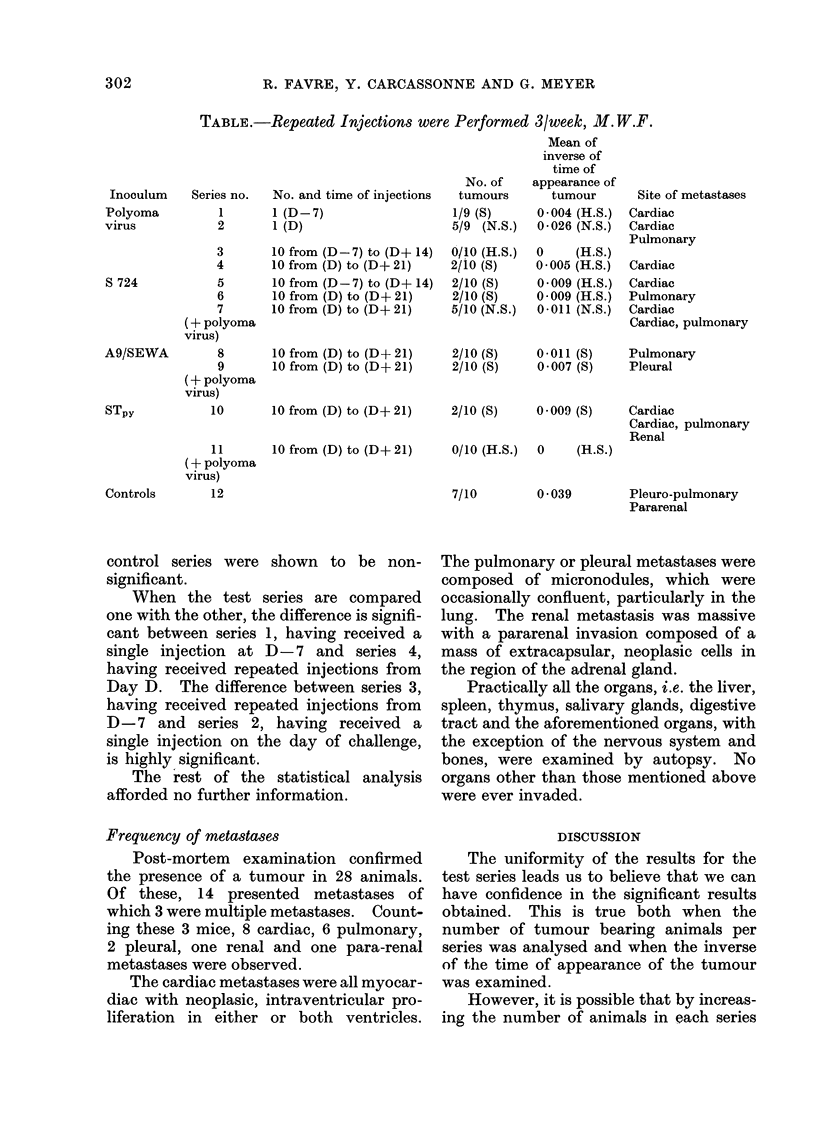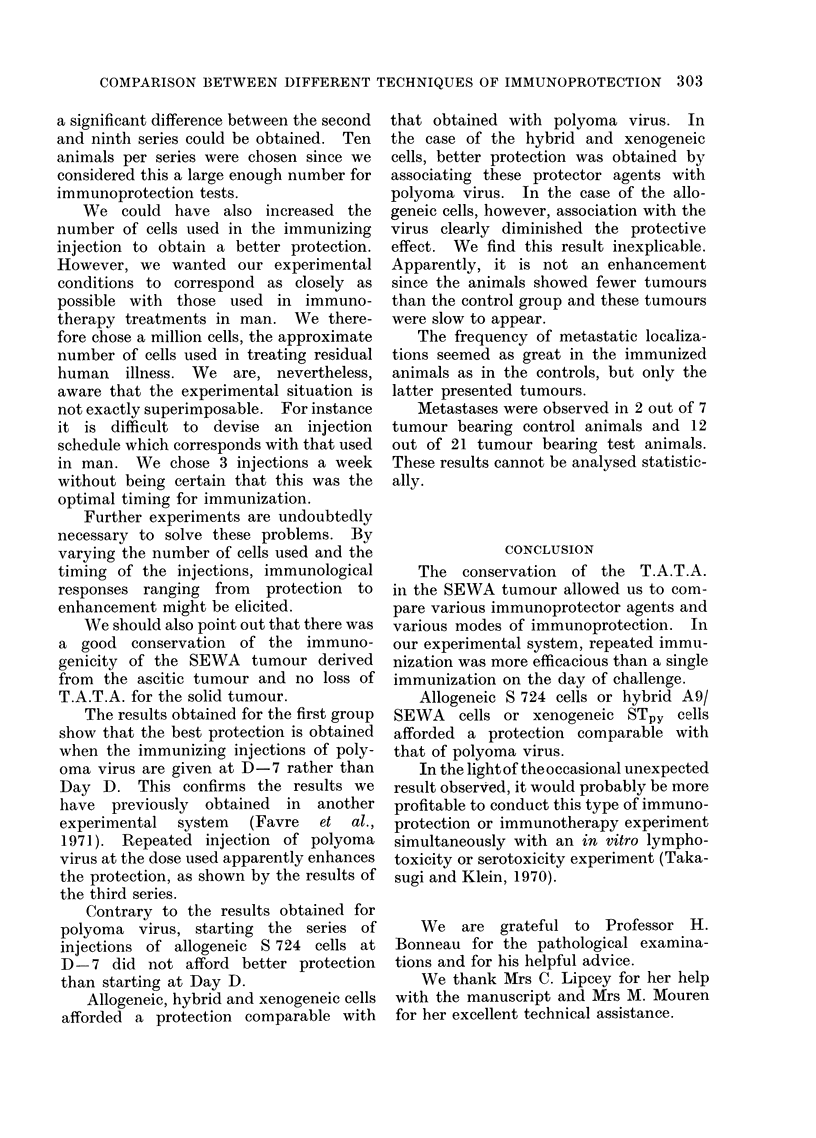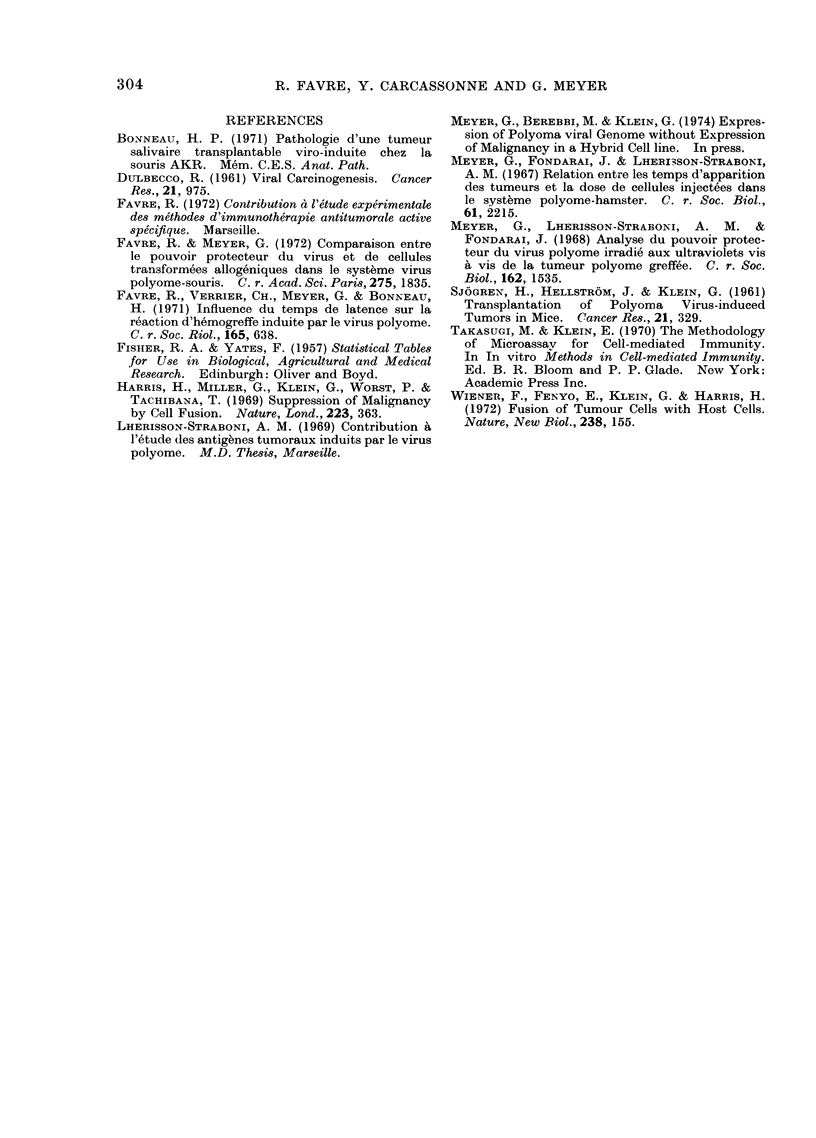# Comparison Between Different Techniques of Immunoprotection in the A-SW Mouse/SEWA Tumour System

**DOI:** 10.1038/bjc.1974.70

**Published:** 1974-04

**Authors:** R. Favre, Y. Carcassonne, G. Meyer

## Abstract

Various procedures for immunoprotection in the A-SW mouse SEWA tumour system were compared. Four types of protector agents were used: polyoma virus, allogeneic S724 cells, A9/SEWA hybrid cells and xenogeneic ST_py_ cells. Several injection schedules were also employed. On the whole, reasonable immunoprotection was obtained. The results, when allogeneic hybrid or xenogeneic cells were used, were comparable with those obtained for polyoma virus. This indicates that the T.A.T.A. of the SEWA tumour had been well conserved. When polyoma virus alone was used as the protector agent, injection 7 days before the tumour challenge, or iterative injection, afforded better protection than a single injection on the day of challenge.


*Br. J. Cancer* (1974) **29**, 300

### COMPARISON BETWEEN DIFFERENT TECHNIQUES OF IMMUNO-PROTECTION IN THE A-SW MOUSE/SEWA TUMOUR SYSTEM

R. FAVRE, Y. CARCASSONNE AND G. MEYER

*From the U.* 119 *de l'I.N.S.E.R.M.*, 27, *bd Leï Roure*, 13009 *Marseille*

Received 16 January 1974. Accepted 16 January 1974

**Summary.—Various procedures for immunoprotection in the A-SW mouse SEWA tumour system were compared. Four types of protector agents were used: polyoma virus, allogeneic S724 cells, A9/SEWA hybrid cells and xenogeneic $ST_{py}$ cells. Several injection schedules were also employed. On the whole, reasonable immunoprotection was obtained. The results, when allogeneic hybrid or xenogeneic cells were used, were comparable with those obtained for polyoma virus. This indicates that the T.A.T.A. of the SEWA tumour had been well conserved. When polyoma virus alone was used as the protector agent, injection 7 days before the tumour challenge, or iterative injection, afforded better protection than a single injection on the day of challenge.**

THE AIM of this study was three-fold. Firstly, we wanted to compare the immunoprotective effect of allogeneic, hybrid and xenogeneic cells in the experimental system of A-SW mouse/polyoma induced tumour cell lines. We have previously shown that cells of C3H mice transformed *in vitro* by polyoma virus afforded a definite immunoprotection in AKR mice against a polyoma induced AKR tumour. The protection by these allogeneic cells was better than that afforded by polyoma virus (Favre and Meyer, 1972).

We also wanted to observe the effect of the time of injection of the polyoma virus, used as the protector agent, on the anti-tumoral protection obtained. Previously, in a study of the kinetics of protection by polyoma virus in the AKR mouse/polyoma tumour system, we showed that the best immunoprotection was obtained when the virus was injected 5 days before the tumour challenge (Favre *et al.*, 1971).

Finally, we wanted to judge the effect of a more intense immunization by repeated injections of polyoma virus. In the polyoma virus-induced tumour/golden hamster system, repetition of the viral injections starting on the day of the tumour challenge (D-day) had led to enhancement (Favre, 1972).

### MATERIALS AND METHODS

*Mice.*—Two and one-half month old syngeneic A-SW mice, bred in our laboratories, were used.

*Tumour cells.*—A solid SEWA line (Sjögren, Hellström and Klein, 1961), transplantable by subcutaneous, dorsal injection was used. This line was derived from a subcutaneous, ventral tumour obtained at the site of injection of ascitic SEWA cells.

Unlike the ascitic SEWA cells which only grow *in vitro* in suspension culture, these cells can be grown on glass. However, they still carry the neo-antigens characteristic of polyoma virus (Meyer, Bérebbi and Klein, 1974). Wiener *et al.* (1972) have shown that these cells are probably hybrid cells between the SEWA cells and the host cells. This possible hybrid character would certainly influence the tumorigenicity of the solid tumour compared with that of the ascitic tumour. In our experiments this is of little importance since all the animals received the same type of tumour.

*Polyoma virus.*—A small-plaque variant of the Toronto strain, produced in secondary mouse embryo cultures, was used (Dulbecco, 1961; Lherisson-Straboni, 1969).

*Allogeneic cells, S724.*—The cells were obtained from a transplantable tumour, S724, derived from a primary tumour of the salivary glands in an AKR mouse (Bonneau, 1971).

*Hybrid cells, A9/SEWA.*—These hybrid cells were provided by Professor G. Klein (Harris *et al.*, 1969).

*Xenogenic cells, $ST_{py}$.*—These cells were obtained from a transplantable tumour, $ST_{py}$ derived from a primary polyoma induced tumour in a golden hamster (Favre, 1972).

One hundred and twenty mice were injected with $10^3$ viable SEWA tumour cells in a 0·5 ml volume at Day D. They were then separated into 12 series of 10. The mice were given 0·5 ml subcutaneous, dorsal injections of either $10^6$ P.F.U. polyoma virus (Py) or a million viable, allogeneic (S 724) or hybrid (A9/SEWA) or xenogenic ($ST_{py}$) cells, or a combination of polyoma virus and the aforementioned viable cells. The exact immunization schedule is shown in the Table.

The mice were examined clinically 3 times a week and autopsied at the end of the experiment or upon death.

The karyotype of the tumours was determined and found to be the same as the karyotype of the injected cells.

### RESULTS

### *Tumour development*

(a) *Incidence of tumours.*—As is shown in the Table, the treated groups developed less tumours than the control groups.

Each series of treated mice were compared statistically both with the control series and with all the other series of treated mice, using the direct method of comparison of proportion, a derivation of Fisher's method (Fisher and Yates, 1957).

The probability (*P*) was calculated by the following formula:

$$P\binom{a}{n};\binom{a_1}{n_1} = \frac{n_1!\ n_2!\ (a_1+a_2)!\ (n_1+n_2-a_1-a_2)!}{a_1!\ (n_1-a_1)!\ a_2!\ (n_2-a_2)!\ (n_1+n_2)!}$$

where a = numerator of the two proportions compared, n = denominator of the two proportions compared.

The significance of the results was thence determined.

Compared with the controls, there was no significant difference for the test series 2 immunized on Day D with polyoma virus and the test series 7 immunized by 10 consecutive, simultaneous injections of polyoma virus and allogeneic cells from Day D. Contrary to this, the difference was either significant, or highly significant, for all the other series when compared with the controls.

When the different series were compared one with the other, the following results were obtained.

For the first 4 series which had received polyoma virus, a significant difference was shown only between series 1 and series 3, injected 7 days before the challenge, on the one hand, and series 2, injected on the day of challenge, on the other.

For the other series, a significant difference was observed only between series 11, injected simultaneously with polyoma virus and xenogeneic $ST_{py}$ cells and series 7, injected simultaneously with polyoma virus and allogeneic S 724 cells.

(b) *Time of appearance of the tumour.*—The significance of the times of appearance of the tumours was also assessed. For statistical purposes, the inverse of the time of appearance was used in the calculations. This allows the inclusion of the non-tumour bearing animals whose inverse times ($1/\infty$) are zero (Meyer, Fondarai and Lherisson-Straboni, 1967; Meyer, Lherisson-Straboni and Fondarai, 1968).

Simple analysis of variance, based on the comparison of variance (Snedecor's test) and subsequent calculation of the F ratios was performed to determine whether the differences observed between two or more series were significant. Values of 95% or 99% were significant or highly significant.

The differences between each of the 9 test series and the control series were found to be significant or highly significant (see Table) confirming the results of section (a). Only the differences between series 2, injected solely with polyoma virus at Day D and series 7, injected with polyoma virus and allogeneic cells, and the

TABLE.—*Repeated Injections were Performed 3/week, M.W.F.*

| Inoculum | Series no. | No. and time of injections | No. of tumours | Mean of inverse of time of appearance of tumour | Site of metastases |
|---|---|---|---|---|---|
| Polyoma virus | 1 | 1 (D−7) | 1/9 (S) | 0·004 (H.S.) | Cardiac |
| | 2 | 1 (D) | 5/9 (N.S.) | 0·026 (N.S.) | Cardiac<br>Pulmonary |
| | 3 | 10 from (D−7) to (D+14) | 0/10 (H.S.) | 0 (H.S.) | |
| | 4 | 10 from (D) to (D+21) | 2/10 (S) | 0·005 (H.S.) | Cardiac |
| S 724 | 5 | 10 from (D−7) to (D+14) | 2/10 (S) | 0·009 (H.S.) | Cardiac |
| | 6 | 10 from (D) to (D+21) | 2/10 (S) | 0·009 (H.S.) | Pulmonary |
| | 7 (+ polyoma virus) | 10 from (D) to (D+21) | 5/10 (N.S.) | 0·011 (N.S.) | Cardiac<br>Cardiac, pulmonary |
| A9/SEWA | 8 | 10 from (D) to (D+21) | 2/10 (S) | 0·011 (S) | Pulmonary |
| | 9 (+ polyoma virus) | 10 from (D) to (D+21) | 2/10 (S) | 0·007 (S) | Pleural |
| $ST_{py}$ | 10 | 10 from (D) to (D+21) | 2/10 (S) | 0·009 (S) | Cardiac<br>Cardiac, pulmonary<br>Renal |
| | 11 (+ polyoma virus) | 10 from (D) to (D+21) | 0/10 (H.S.) | 0 (H.S.) | |
| Controls | 12 | | 7/10 | 0·039 | Pleuro-pulmonary<br>Pararenal |

control series were shown to be non-significant.

When the test series are compared one with the other, the difference is significant between series 1, having received a single injection at D−7 and series 4, having received repeated injections from Day D. The difference between series 3, having received repeated injections from D−7 and series 2, having received a single injection on the day of challenge, is highly significant.

The rest of the statistical analysis afforded no further information.

*Frequency of metastases*

Post-mortem examination confirmed the presence of a tumour in 28 animals. Of these, 14 presented metastases of which 3 were multiple metastases. Counting these 3 mice, 8 cardiac, 6 pulmonary, 2 pleural, one renal and one para-renal metastases were observed.

The cardiac metastases were all myocardiac with neoplasic, intraventricular proliferation in either or both ventricles. The pulmonary or pleural metastases were composed of micronodules, which were occasionally confluent, particularly in the lung. The renal metastasis was massive with a pararenal invasion composed of a mass of extracapsular, neoplasic cells in the region of the adrenal gland.

Practically all the organs, *i.e.* the liver, spleen, thymus, salivary glands, digestive tract and the aforementioned organs, with the exception of the nervous system and bones, were examined by autopsy. No organs other than those mentioned above were ever invaded.

### DISCUSSION

The uniformity of the results for the test series leads us to believe that we can have confidence in the significant results obtained. This is true both when the number of tumour bearing animals per series was analysed and when the inverse of the time of appearance of the tumour was examined.

However, it is possible that by increasing the number of animals in each series a significant difference between the second and ninth series could be obtained. Ten animals per series were chosen since we considered this a large enough number for immunoprotection tests.

We could have also increased the number of cells used in the immunizing injection to obtain a better protection. However, we wanted our experimental conditions to correspond as closely as possible with those used in immunotherapy treatments in man. We therefore chose a million cells, the approximate number of cells used in treating residual human illness. We are, nevertheless, aware that the experimental situation is not exactly superimposable. For instance it is difficult to devise an injection schedule which corresponds with that used in man. We chose 3 injections a week without being certain that this was the optimal timing for immunization.

Further experiments are undoubtedly necessary to solve these problems. By varying the number of cells used and the timing of the injections, immunological responses ranging from protection to enhancement might be elicited.

We should also point out that there was a good conservation of the immunogenicity of the SEWA tumour derived from the ascitic tumour and no loss of T.A.T.A. for the solid tumour.

The results obtained for the first group show that the best protection is obtained when the immunizing injections of polyoma virus are given at D—7 rather than Day D. This confirms the results we have previously obtained in another experimental system (Favre *et al.*, 1971). Repeated injection of polyoma virus at the dose used apparently enhances the protection, as shown by the results of the third series.

Contrary to the results obtained for polyoma virus, starting the series of injections of allogeneic S 724 cells at D—7 did not afford better protection than starting at Day D.

Allogeneic, hybrid and xenogeneic cells afforded a protection comparable with that obtained with polyoma virus. In the case of the hybrid and xenogeneic cells, better protection was obtained by associating these protector agents with polyoma virus. In the case of the allogeneic cells, however, association with the virus clearly diminished the protective effect. We find this result inexplicable. Apparently, it is not an enhancement since the animals showed fewer tumours than the control group and these tumours were slow to appear.

The frequency of metastatic localizations seemed as great in the immunized animals as in the controls, but only the latter presented tumours.

Metastases were observed in 2 out of 7 tumour bearing control animals and 12 out of 21 tumour bearing test animals. These results cannot be analysed statistically.

### CONCLUSION

The conservation of the T.A.T.A. in the SEWA tumour allowed us to compare various immunoprotector agents and various modes of immunoprotection. In our experimental system, repeated immunization was more efficacious than a single immunization on the day of challenge.

Allogeneic S 724 cells or hybrid A9/SEWA cells or xenogeneic $ST_{py}$ cells afforded a protection comparable with that of polyoma virus.

In the light of the occasional unexpected result observed, it would probably be more profitable to conduct this type of immunoprotection or immunotherapy experiment simultaneously with an *in vitro* lymphotoxicity or serotoxicity experiment (Takasugi and Klein, 1970).

We are grateful to Professor H. Bonneau for the pathological examinations and for his helpful advice.

We thank Mrs C. Lipcey for her help with the manuscript and Mrs M. Mouren for her excellent technical assistance.

### REFERENCES

BONNEAU, H. P. (1971) Pathologie d'une tumeur salivaire transplantable viro-induite chez la souris AKR. Mém. C.E.S. *Anat. Path.*

DULBECCO, R. (1961) Viral Carcinogenesis. *Cancer Res.*, **21**, 975.

FAVRE, R. (1972) *Contribution à l'étude expérimentale des méthodes d'immunothérapie antitumorale active spécifique.* Marseille.

FAVRE, R. & MEYER, G. (1972) Comparaison entre le pouvoir protecteur du virus et de cellules transformées allogéniques dans le système virus polyome-souris. *C. r. Acad. Sci. Paris*, **275**, 1835.

FAVRE, R., VERRIER, CH., MEYER, G. & BONNEAU, H. (1971) Influence du temps de latence sur la réaction d'hémogreffe induite par le virus polyome. *C. r. Soc. Riol.*, **165**, 638.

FISHER, R. A. & YATES, F. (1957) *Statistical Tables for Use in Biological, Agricultural and Medical Research.* Edinburgh: Oliver and Boyd.

HARRIS, H., MILLER, G., KLEIN, G., WORST, P. & TACHIBANA, T. (1969) Suppression of Malignancy by Cell Fusion. *Nature, Lond.*, **223**, 363.

LHERISSON-STRABONI, A. M. (1969) Contribution à l'étude des antigènes tumoraux induits par le virus polyome. *M.D. Thesis, Marseille.*

MEYER, G., BEREBBI, M. & KLEIN, G. (1974) Expression of Polyoma viral Genome without Expression of Malignancy in a Hybrid Cell line. In press.

MEYER, G., FONDARAI, J. & LHERISSON-STRABONI, A. M. (1967) Relation entre les temps d'apparition des tumeurs et la dose de cellules injectées dans le système polyome-hamster. *C. r. Soc. Biol.*, **61**, 2215.

MEYER, G., LHERISSON-STRABONI, A. M. & FONDARAI, J. (1968) Analyse du pouvoir protecteur du virus polyome irradié aux ultraviolets vis à vis de la tumeur polyome greffée. *C. r. Soc. Biol.*, **162**, 1535.

SJÖGREN, H., HELLSTRÖM, J. & KLEIN, G. (1961) Transplantation of Polyoma Virus-induced Tumors in Mice. *Cancer Res.*, **21**, 329.

TAKASUGI, M. & KLEIN, E. (1970) The Methodology of Microassay for Cell-mediated Immunity. In In vitro *Methods in Cell-mediated Immunity.* Ed. B. R. Bloom and P. P. Glade. New York: Academic Press Inc.

WIENER, F., FENYO, E., KLEIN, G. & HARRIS, H. (1972) Fusion of Tumour Cells with Host Cells. *Nature, New Biol.*, **238**, 155.